# Effect of P_2_O_5_ Content on Luminescence of Reduced Graphene-Oxide-Doped ZnO–P_2_O_5_ Nano-Structured Films Prepared via the Sol–Gel Method

**DOI:** 10.3390/ma16186156

**Published:** 2023-09-11

**Authors:** Ileana Cristina Vasiliu, Ana Violeta Filip, Irinela Chilibon, Mihail Elisa, Cristina Bartha, Victor Kuncser, Aurel Leca, Lucica Boroica, Bogdan Alexandru Sava, Roxana Trusca, Mihai Eftimie, Adrian Nicoara

**Affiliations:** 1 National Institute for Research and Development in Optoelectronics—INOE 2000, 409 Atomistilor Str., RO-077125 Magurele, Romania; icvasiliu@inoe.ro (I.C.V.); elisa@inoe.ro (M.E.); 2 National Institute for Laser, Plasma and Radiation Physics (INFLPR), 409 Atomistilor Str., RO-077125 Magurele, Romania; boroica_lucica@yahoo.com (L.B.); bogdan.sava@inflpr.ro (B.A.S.); 3 National Institute of Materials Physics, 405 A Atomistilor Str., RO-077125 Magurele, Romania; cristina.bartha@infim.ro (C.B.); kuncser@infim.ro (V.K.); aurel.leca@infim.ro (A.L.); 4 Department of Science and Engineering of Oxide Materials and Nanomaterials, National University of Science and Technology “Politehnica” of Bucharest, 1 Polizu Street, RO-011061 Bucharest, Romania; mihai.eftimie@upb.ro (M.E.); adrian.nicoara@upb.ro (A.N.); 5 Faculty of Engineering in Foreign Languages, University of Science and Technology “Politehnica” of Bucharest, 313 Splaiul Independentei Str., RO-060042 Bucharest, Romania; truscaroxana@yahoo.com

**Keywords:** ZnO–P_2_O_5_–rGO nanostructures, tuned fluorescence, bandgap engineering, sol–gel

## Abstract

A convenient and low-cost sol–gel approach for the one-step synthesis of ZnO–P_2_O_5_–rGO nanostructures with tuned bandgap and fluorescence was investigated. The obtained hybrid nanostructures exploit the properties of zinc oxide, graphene oxide and phosphorous oxide as promising candidates for a wide range of optoelectronic applications. A predominant amorphous structure, ZnO–P_2_O_5_–rGO, containing ZnO nanorods was evidenced by X-ray diffraction analysis (XRD) and scanning electron microscopy (SEM). The estimated size of the ZnO nanorods in nanostructures with P_2_O_5_ was noticed to decrease when the P_2_O_5_/ZnO ratio was increased. The presence of ZnO, P_2_O_5_ and rGO was confirmed by Fourier-transform infrared spectroscopy (FTIR) and Raman investigation. P_2_O_5_ was noticed to tune the bandgap and the fluorescence emissions of the nanostructured films, as estimated by UV–Vis–NIR and fluorescence spectroscopy, respectively. The electrical measurements performed at room temperature showed that the main influence on the film’s resistivity does not come from the 1% rGO doping but from the P_2_O_5_/ZnO ratio. It was found that a 10/90 molar ratio of P_2_O_5_/ZnO decreases the resistivity almost seven-fold compared with rGO-doped ZnO films.

## 1. Introduction

Nowadays, optical materials, including semiconductors, are widely fabricated into thin films, which are essential for optoelectronic and microelectronic devices such as sensors, lighting devices and solar cells. As a consequence of the remarkable progress in nanotechnologies, the structure of the thin films can be further engineered at different scales to enhance the optical performance of the materials, which is attributed to the enhancement of the interaction between the light waves and these nanostructured thin films. Recently, zinc oxide (ZnO)-based nanostructures have been intensively studied due to their interesting new properties.

ZnO belongs to the wurtzite family of structures with the three fastest growth directions, <0001>, <0110> and <2110>, and polar-surface-induced phenomena, with a direct wide band gap of 3.37 eV and a large excitation binding energy (60 meV), exhibiting near-ultraviolet emission and transparent conductivity. As an oxide with noncentral symmetry, ZnO is piezoelectric, a property that qualifies it for use in building electromechanical-coupled sensors and transducers. ZnO is also bio-safe and biocompatible, and can be used for biomedical applications without coating.

The ZnO–graphene heterostructure nanohybrids integrate the physical properties of graphene and ZnO, providing a unique platform for the exploration of a wide variety of applications [1] ranging from photodetectors [2,3] and gas sensors [4] to stress/strain sensors [5,6], lasers [7] and piezoelectric nanogenerators [8], to mention just a few that have recently been reported.

The preparation of a composite structure of ZnO with graphene or graphene oxide is an efficient way to control the morphology, band gap and surface-defection states of ZnO nanostructures [9,10].

Nanostructured ZnO–carbon materials are an emerging class of nanostructures with unique optical properties, including photoluminescence. ZnO photoluminescence emissions in the UV and visible regions depend on the synthesis routes, shape, size, deep level and surface defects. Carbon nanomaterials such as graphene oxide (GO) and reduced graphene oxide (rGO) can modify the surface defects in ZnO, allowing the tuning of these photoluminescence properties in order to produce, for example, white light. The efficient energy transfer from the ZnO to carbon nanostructures makes these nanostructures suitable candidates not only for energy-harvesting applications but also for use as biosensors, photodetectors and low-temperature thermal imaging. The literature reported the preparation of ZnO–graphene oxide using the hydrothermal method [11,12], via the combination of a simple hydrothermal reaction and spray drying [13], via the radiofrequency magnetron sputtering technique [14] and via ultrasonic-assisted spray pyrolysis [15]. Graphene-assisted controlled growth of highly aligned ZnO nanorods and nanoribbons using few layers of graphene prepared by chemical vapor deposition (CVD) [16] and the fabrication of ZnO–GO composite films by laser technique were also reported [17]. Doped ZnO with carbon-based materials prepared via the sol–gel method with advantages such as low temperature synthesis, large-scale productivity and cost effectiveness were reported recently [18,19,20,21,22].

J.Wang reported on the use of surface-modified zinc phosphate (Zn_3_(PO_4_)_2_) nanocrystals widely applied in the coating industry, medicine, electrical fields, particularly for their anti-corrosion properties due to low solubility in a water/biological environment prepared via the ultrasonic–template–microwave (UTM)-assisted route [23].

The present research work aimed at investigating sol–gel methods as a convenient and low-cost approach for the one-step synthesis of ZnO–P_2_O_5_–rGO nanostructures with bandgap engineering, and tuned fluorescence. The investigated materials exploit the properties of hybrid nanostructures based on zinc oxide, graphene oxide and phosphorous oxide, exploring their suitability for a wide range of optoelectronic applications.

## 2. Materials and Methods

### 2.1. Zinc Phosphate Graphene-Doped Nanostructured Films Preparation

The following chemicals were used for sol–gel synthesis as received without previous purification: zinc acetylacetonate (Zn(C_5_H_7_O_2_)_2_·1H2O, 99.98 wt.% Sigma Aldrich), monoethanolamine (MEA) (C_2_H_7_NO, Alfa Aesar), triethyl phosphate (TEP) (C_6_H_15_O_4_P, 99.8 wt.%, Sigma Aldrich), distilled water, and ethanol (C_2_H_5_OH, 96 ± 2 wt.%, Alfa Aesar, Haverhill, MA, USA). An 18 mg/mL solution of reduced graphene oxide (rGO) in ethanol was prepared as previously reported [24]. ISOLAB microscope slides were used as substrates after cleaning by washing several times with ethanol and deionized water and drying at room temperature.

A stock solution was prepared by dissolving zinc acetylacetonate in ethanol at room temperature, followed by the addition of MEA in a ratio of 1 mol MEA: 1 mol zinc acetylacetonate (MEA/ZnAc = 1). Appropriate amounts of rGO suspension and TEP were added, as presented in Table 1, and the resulting sol was aged at room temperature for 72 h under continuous magnetic stirring. The molar ratios of ZnO/P_2_O_5_ were 90:10, 86:14 and 80:20, as presented in Table 1.

The sols formed were deposited on the glass substrate by spin coating at a rotation rate of 2000 rpm at room temperature. Films of 1, 10 and 20 layers were deposited. After each spin-coated layer, the samples were placed on a hot plate at 80 °C for two minutes. After the 1-, 10- and 20-layer deposition, the samples were dried at 200 °C (5 °C/h) for 30 min, followed by thermal treatment at 400 °C for 30 min.

### 2.2. Investigating Methods

X-ray diffraction analysis was performed using a BRUKER D8 ADVANCE (Billerica, MA, USA)- type X-ray diffractometer (CuKα, λ = 1.5405 Å). The X-ray data were acquired at room temperature using a step of 0.020° and 5 s integration time. All samples were scanned between 5° and 70° (2θ range). The ICDD powder diffraction database was used for phase identification [25].

Fourier-transform infrared (FTIR) spectra were recorded using a Perkin Elmer Spectrophotometer-Spectrum 100 provided with the Universal Attenuated Total Reflectance (UATR) accessory, in the range of 400–4000 cm^−1^, with 4 cm^−1^ resolution, 20 total scans and a measurement error of ±0.1%.

Optical properties were measured with a Spectrophotometer Lambda 1050. (PerkinElmer, Waltham, MA, USA) in the UV–Vis–NIR domain in the range of 320–2500 nm, with a measurement error of ±0.03%.

The morphology and elemental compositions of the samples were studied with the aid of a scanning electron microscopy (SEM) with energy-dispersive X-ray (EDX) analysis using a FEI Inspect F50 system (FEI Europe B.V. Eindhoven, The Netherlands).

The luminescence spectra were collected with a spectrofluorometer FluoroLog-3, HORIBA Jobin Yvone S.A.S. (Paris, France) in the range of 850–1600 nm, using an 850 nm excitation wavelength from a Xe lamp of 450 W, with a measurement error of ±0.5 nm.

Raman spectra were collected by means of a LabRam HR Evolution HORIBA (Palaiseau, France), acquisition time 2 s, accumulation 20, laser 514 nm, hole diameter 100 micro, objective 50×, grating 600 gr/mm, ND filter 100%, range 100–16,000 cm^−1^, with a measurement error of ±0.5 cm^−1^.

Electrical measurements were performed at room temperature (RT) using a Keithley 2450 Source Meter. Two-point geometry was considered using pairs of linear electrodes placed at different distances between 0.5 and 3 mm. Rectangular pieces of samples (between 2.6 and 3.5 mm wide) were used with the linear contacts crossing the entirety of the sample width. Next, 10 nA currents were applied under potential drops of of tens of Volts.

## 3. Results and Discussion

### 3.1. X-ray Diffraction (XRD) Investigations

**Figure 1:**
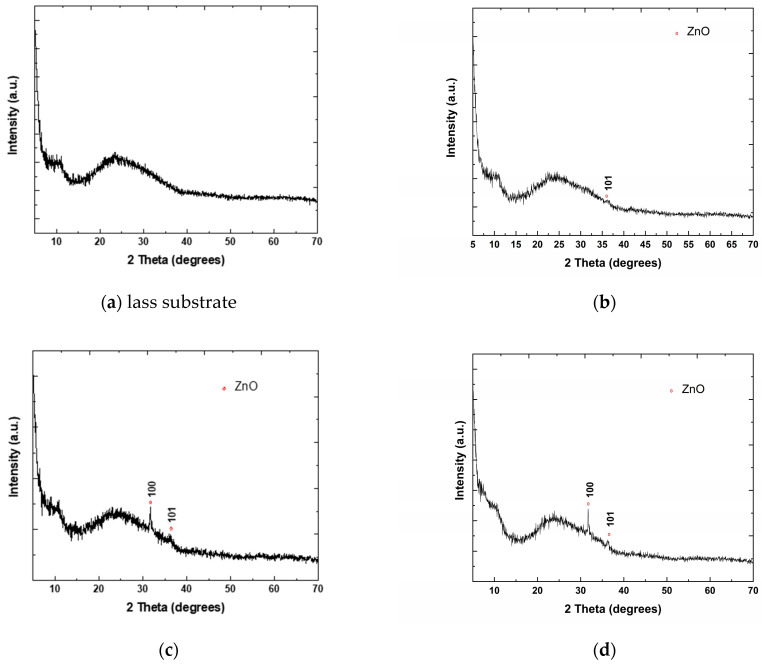

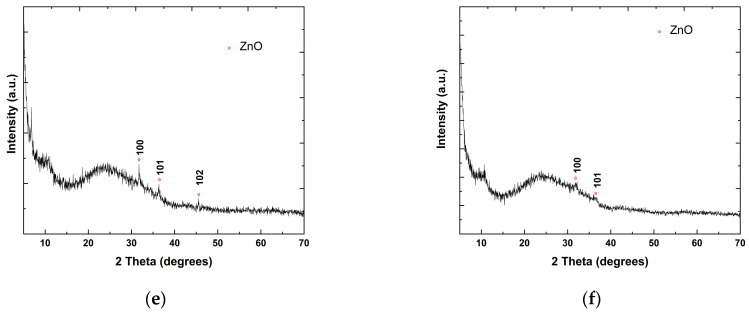
XRD patterns of analyzed samples: (**a**) glass substrate; (**b**) ZnO 20 layers; (**c**) 80ZnO20P_2_O_5_rGO 20 layers; (**d**) 86ZnO14P_2_O_5_rGO 20 layers; (**e**) 90ZnO10P_2_O_5_rGO 20 layers; (**f**) ZnOrGO 20 layers.

The XRD patterns of all analyzed samples are presented in Figure 1a–f. It was noticed that three samples (Figure 1a,b,f) out of the six analyzed have an amorphous character, similar to that of the glass substrate (Figure 1a). In these samples, the amount of crystallized ZnO is greatly reduced (negligible narrow diffraction peaks specific to a crystalline phase), almost below the detection limit of the device. For the samples with P_2_O_5_ content, a hexagonal phase of ZnO (space group P63mc) was identified according to PDF card 01-080-6503. The crystallite size was estimated for the samples (c) 80ZnO20P_2_O_5_rGO 20 layers, (d) 86ZnO14P_2_O_5_rGO-20 layers and (e) 90ZnO10P_2_O_5_rGO 20 layers using the Scherrer formula [26] applied to the (100) reflections corresponding to the three samples. The estimated sizes were around 10.34 nm for sample (c), 8.5 nm for sample (d) and 18 nm for sample (e), respectively. These values are very close to those of the nanorods’ diameters obtained via the SEM investigations of these samples.

### 3.2. Fourier-Transform Infrared Spectroscopy (FTIR) Investigations

**Figure 2:**
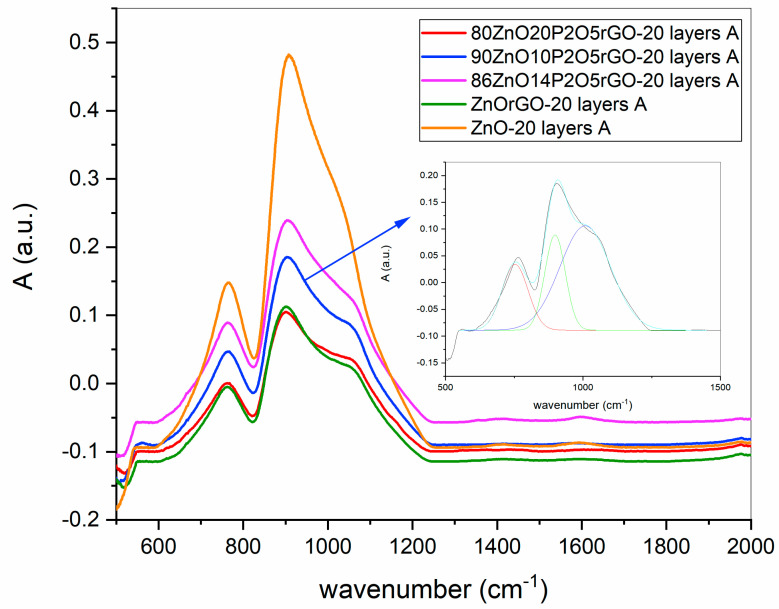
FTIR spectra for ZnO-based nanostructured films; the deconvoluted spectra of the sample 90ZnO10P_2_O_5_rGO in insertion.

The FTIR absorption spectra of all samples are presented in Figure 2, and the vibration bands after deconvolution, together with their assessments, are shown in Table 2. The vibrational band noticed at 546 cm^−1^ in all samples was assigned to the Zn–O stretching mode and confirms the formation of ZnO [27]. The vibration modes of the silica-based glass substrate with broad bands centered at around 760 and 910 cm^−1^ and assigned to the Si-O- (stretching mode) in the range 750–760 cm^−1^ and 890–975 cm^−1^ were present in all the samples. The information from the 1 mm thickness of the glass substrate overlapped with the information received from the films with a thickness of only hundreds of nanometers [28]. However, in some samples, new shoulders were noticed, and after deconvolution, these new bands, identified in all samples, containing rGO and P_2_O_5_ at 988–1010 cm^−1^, were ascribed to the overlapping of the Si-O vibrational modes with C-O-C stretching (epoxy group) [29] and with υs Si-O-P in samples containing phosphorous [30,31].

### 3.3. UV–VIS–NIR Spectroscopy Investigations

**Figure 3:**
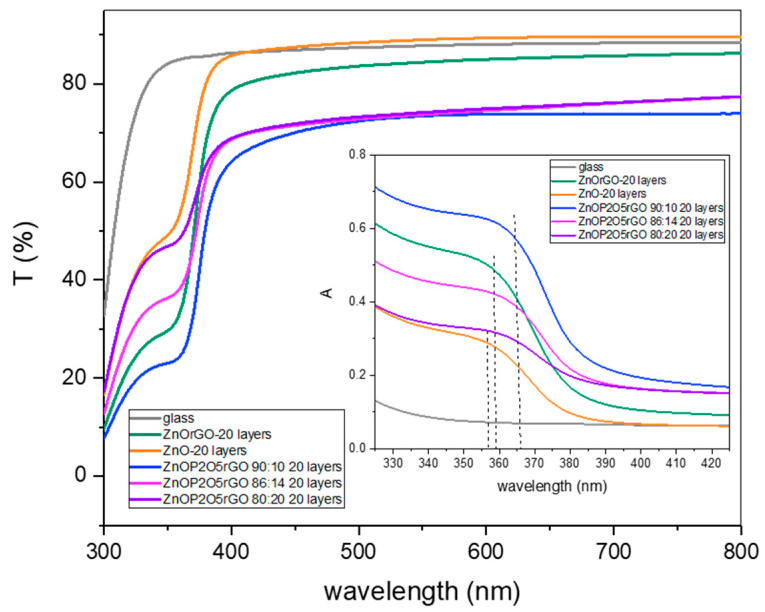
UV–VIS–NIR spectra of ZnO, ZnOrGO and ZnOrGOP_2_O_5_ films (the absorbance spectra in insertion).

The UV–VIS–NIR transmission and absorption spectra (insert) of ZnO, ZnO–graphene hybrid and ZnO–P_2_O_5_–graphene hybrids were recorded, and the results are presented in Figure 3. For the pure ZnO-layered structure, the characteristic absorption band attributed to the excitonic absorptions centered at 356 nm was noticed. The reduced graphene oxide in ZnO/rGO nanocomposites slightly shifted the absorption band edge to 358 nm, together with a decreased transmission from 89% to 85% in the visible region compared with the ZnO nanolayer, suggesting an increase in the surface electric charge of ZnO due to the graphene that may modify the exciton formation [32,33,34]. P_2_O_5_ red-shifted the excitonic absorption edges to 360–365 nm, together with a decreased transmission from 85–89% to 74–77% for the visible domain, as compared with the samples without phosphorous content, indicating a chemical interaction of P_2_O_5_ with the ZnO–rGO matrix and also an effective integration of the materials. However, the transmittance in the visible domain was greater than 75% for all samples.

**Figure 4:**
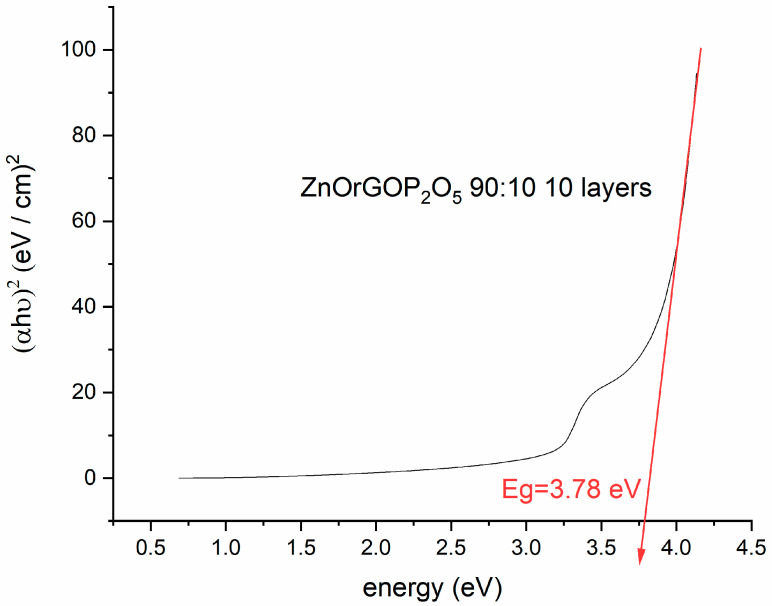
Bandgap estimation of 90ZnO10P_2_O_5_rGO film by Tauc plot method.

**Figure 5:**
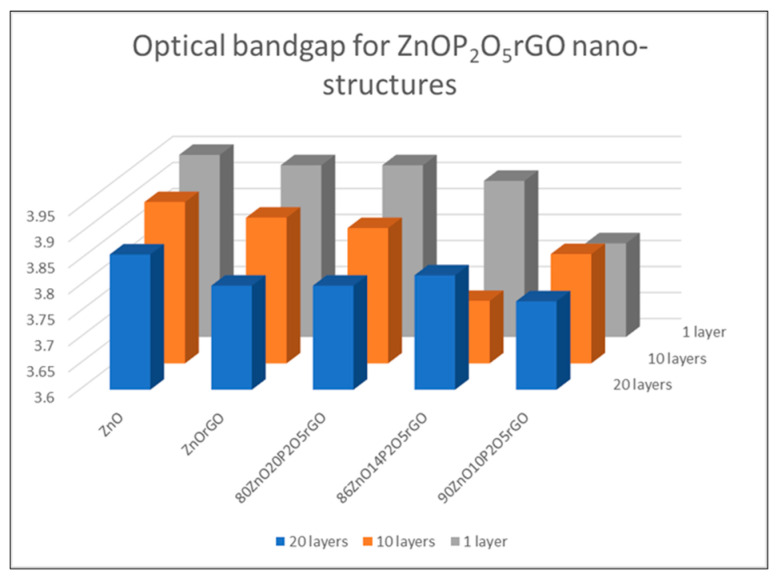
Optical bandgap of ZnOP_2_O_5_rGO nanostructures.

The direct bandgaps of nanocomposites structures were estimated from the graph of hν versus (αhν)^2^, for the absorption coefficient α that is related to the bandgap Eg as (αhν)^2^ = k (hν − Eg), where hν is the incident light energy and k is a constant [35]. An example of the graphical estimation of the bandgap for 90ZnO10P_2_O5rGO is presented in Figure 4. Figure 5 presents the variation in the optical bandgap of the zinc-based nanostructures as a function of P_2_O_5_ content and the number of deposited layers.

The band gaps decreased as a function of rGO and phosphorous oxide contents with a minimum of 3.77 eV for the film 90ZnO10P_2_O_5_rGO.The optical band gap (Eg) was found to be dependent on the number of layers, as expected [36,37]. A decrease in the band gap energy of the structures with an increase in the number of layers was noticed for the ZnO, ZnOrGO and 80ZnO20P_2_O_5_rGO samples because of the higher carrier concentration in the conduction and valence bands, which led to enhanced carrier–carrier interaction. For the samples 86ZnO14P_2_O_5_rGO and 90ZnO10P_2_O_5_rGO, apart from the dependence of the absorption band edge on the size of the constituent nanocrystals, P_2_O_5_ also contributes to the distortion of some of the crystal lattice and consequently affects the crystal size and lattice. Since the optical properties strongly depend on the micro-structure of the materials, we found that P_2_O_5_ can tune the bandgap of ZnO-based films and, consequently, can tune the optical properties.

### 3.4. SEM Spectroscopy Investigations

**Figure 6:**
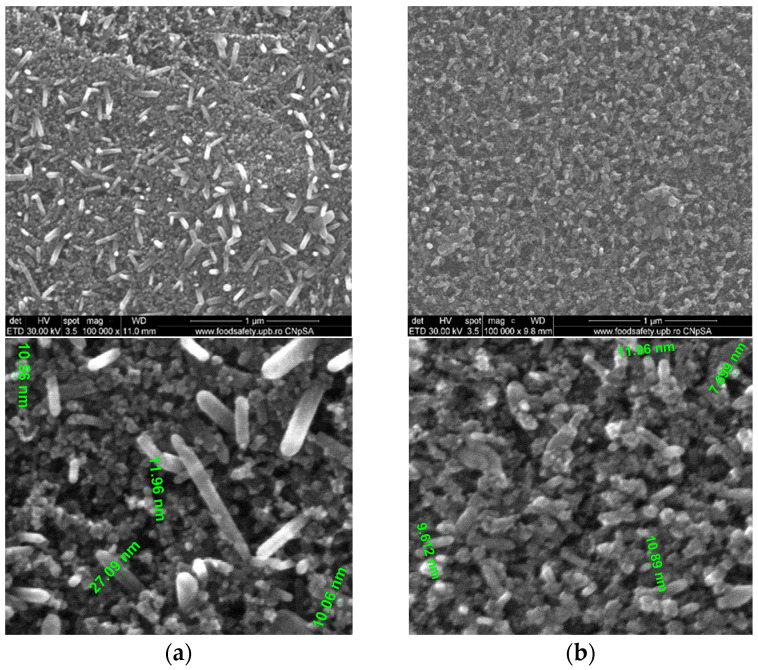

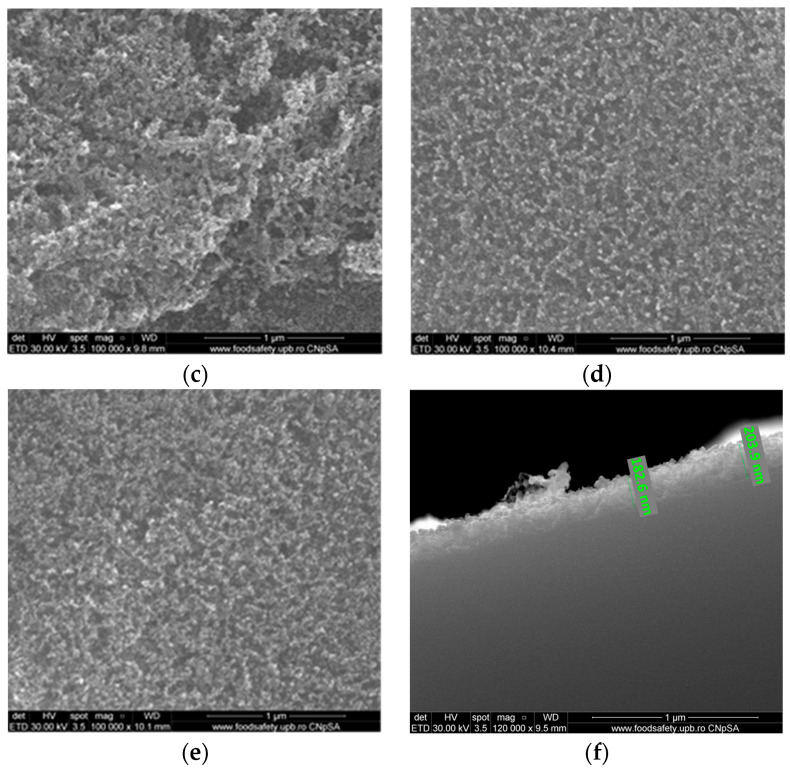
SEM images of the sol–gel 20-layered films: (**a**) 90ZnO10P_2_O_5_rGO and detailed image with crystalites’size; (**b**) 86ZnO14P_2_O_5_rGO and detailed image with crystalites’size; (**c**) 80ZnO20P_2_O_5_rGO; (**d**) ZnOrGO; (**e**) ZnO; (**f**) typical thickness of a ZnOP_2_O_5_rGO film.

SEM images of the typical 20-layered films are presented in Figure 6a–e.

Figure 6a–e presents the typical SEM images of the deposited sol–gel films with 20 layers. ZnO crystallites as nanorods were noticed in all samples. The distribution of the diameter of the crystallites in the samples varies in the interval of 5–10 nm for samples ZnO, ZnOrGO and 80ZnO20P_2_O_5_rGO. Samples with molar ratios of 86/14 and 90/10 ZnO/P_2_O_5_ displayed an increase in the diameter of the nanorods up to 20 and 26 nm, respectively, and an increased distribution of the nanorods with diameters of around 10 nm. These observations are in agreement with the estimated sizes from XRD investigations.

The 200 nm typical thickness of a film containing ZnO, P_2_O_5_ and rGO with 20 consecutively deposited layers is presented in Figure 6f.

### 3.5. Photoluminescence Spectroscopy

**Figure 7:**
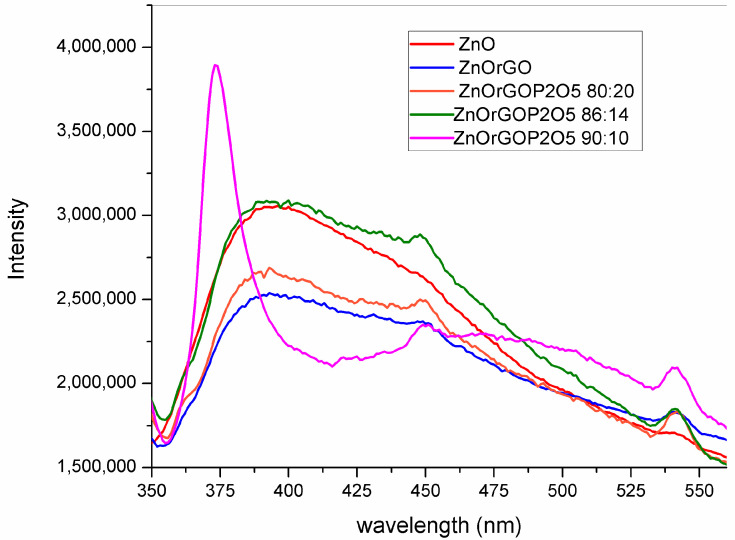
Photoluminescence (PL) spectra of the sol–gel 20-layered films: 90ZnO10P_2_O_5_rGO; 86ZnO14P_2_O_5_rGO; 80ZnO20P_2_O_5_rGO; ZnOrGO; ZnO.

The PL spectra of the prepared ZnO-based nanostructured composite films, recorded at room temperature with a 330 nm excitation wavelength, are presented in Figure 7.

Three spectrally distinct PL bands were noticed, namely the high sharp intensity band (UV emission) with maximum levels centered at 374 nm (recorded for sample 90ZnOrGO10P_2_O_5_) and the broad band centered at 393 nm (recorded for samples ZnO, ZnOrGO, 86ZnOrGO14P_2_O_5_ and 80ZnOrGO20P_2_O_5_), the medium-intensity broad band (blue emission) centered at 448 nm for all samples and the lowest-intensity and sharp band (green emission) at 542 nm displayed for all samples except for ZnO.

The near-band-edge (NBE) UV emission was associated with the radiative recombination of free excitons in ZnO. The visible emission bands were attributed to surface defects, such as oxygen vacancies (VO) (or zinc dangling bonds) and zinc vacancies (VZn) (or oxygen dangling bonds) [12,38].

The presence of defects on the surfaces of ZnO films may result in significant charge recombination rates. Especially the solution processing of ZnO films often leads to a high density of surface defects that can act as recombination centers for photogenerated charge carriers, decreasing the photocurrent and power conversion efficiency [38].

Our prepared 10% molar of P_2_O_5_ and 1% rGO in 90ZnOrGO10P_2_O_5_ sample tuned the fluorescence properties. The enhanced NBE intensity, together with a quenching of the visible emissions by passivating the surface defects with simultaneous enhancement of the conductivity of ZnO, was achieved within one sol–gel stage of synthesis. Other reported fluorescence tuning methods, such as ZnO thin-film treatment via surface covering with a thin ZnS coating [39] or by fluorine plasma treatment [40], consist of at least two stages apart from the temperature treatment. Polydorou et al. [38] reported an increase in the near-band edge (NBE) intensity and a reduction in the broadband intensity in the visible range upon ZnO thin film treatment in pure and mixed SF6 plasma with subsequent improvements in device efficiency and lifetime of inverted polymer solar cells.

For all prepared sol–gel samples containing graphene (Figure 7), as shown by the PL spectra, the luminescence was substantially quenched after the addition of rGO due to the interfacial charge transfer between ZnO nanorods and rGO layers.

The blue emission at 448 nm was attributed to the electron transition from a shallow donor level of zinc interstitials (Zni) to the top of the valence band (VB) [41]. The green emission peak observed around 540 nm is due to the transitions from the oxygen vacancy (VO) level of ZnO NRs [42]. The presence of intrinsic defects in the ZnO lattice structure causes photoemissions in the green or yellow/orange/red spectral regime. Green emissions are often attributed to singly ionized oxygen vacancies, or oxygen vacancies and zinc interstitials.

Blue and green emissions of the sol–gel prepared ZnO films, as presented in PL spectra, were not noticed, probably due to the predominant amorphous phase of the film.

### 3.6. Raman Analysis

**Figure 8:**
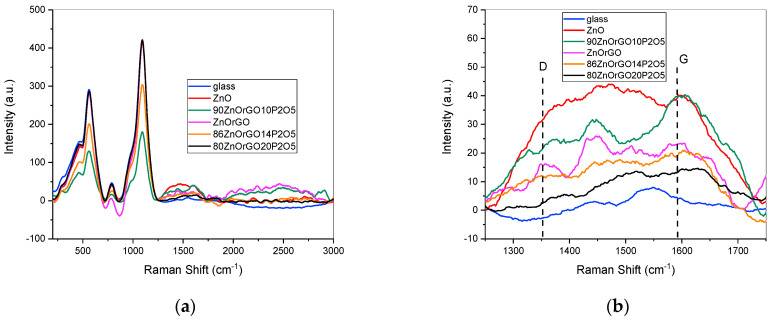
Raman spectra of the sol–gel 20 layered films: 90ZnO10P_2_O_5_rGO, 86ZnO14P_2_O_5_rGO, 80ZnO20P_2_O_5_rGO, ZnOrGO, ZnO; wide range (**a**) and detail (**b**).

We have collected the Raman spectra for all samples, attempting to estimate the crystallite symmetry and prove the existence of graphene oxide in the sol–gel films. Figure 8a,b shows the Raman spectra of the thin films in a wide range (450–3000 cm^−1^) and on a reduced interval (1250–1750 cm^−1^), respectively. The ZnO Raman characteristic peaks from ~437, 570, 790 and 1100 cm^−1^ [43] could not be noticed in any of the samples due to the overlapping with the strong contribution of the glass substrate.

The wide band of the ZnO film (Figure 8b) in the interval 1250–1750 cm^−1^ could be due to the light scattering in amorphous structures, which was predominant in our films, in accordance with the XRD patterns. However, according to the reported literature, the carbon defects in the structures of these films should not be excluded since C is always present as an unintentional dopant introduced during the growth of ZnO by the sol–gel method, which is based on organic precursor compounds [43,44].

Different features were noticed for the Raman spectra of the samples containing rGO. It is well known that GO exhibits two main characteristic peaks: a D band located at ~1350 cm^−1^, and a G band located at ~1590 cm^−1^. The D band refers to some disorders and defects in the graphitic structure, while the G band refers to the in-plane vibration of sp2 carbon-type structures [45,46].

The D band appears in samples ZnOrGO and 90ZnOrGO10P_2_O_5_, while the G band was noticed in all samples, which suggests the presence of the reduced graphene oxide material in the ZnO–rGO nanostructures.

The intensity ratio of the D band (ID) to the G band (IG) correlated with the disorder evaluation in carbonaceous materials could not be estimated due to the complexity of the system and the presence of ZnO. However, the Raman investigations proved the existence of rGO in the nanostructured thin films.

### 3.7. Electrical Measurements

The average resistance value obtained from different contact pairs on each film, in the case of the analyzed 20-layered films, is presented in Table 3.

The resistivity of each film was computed according to the formula: *ρ = R∙t∙w/l*, where *R* is the resistance, *t* is the film thickness, *w* is the film width (equating the wideness of the rectangular sample) and *l* is the distance between the pair of electrodes. The thickness of all films was approximated at 200 nm; the sample/film widths are mentioned in Table 3. A relatively low dispersion (i.e., below 10%) of the resistivity values was obtained, with resistances collected from different contact pairs, with a slightly decreasing trend at increasing distances between contacts. This observation gives support for a reasonable homogeneous resistivity of the films over the mm range. As a direct consequence, an average resistivity can be calculated by using the average value of the resistance and the corresponding average distance between the considered contact pairs, as mentioned in Table 3.

It can be observed that the main influence on the resistivity does not come from the 1 wt. % of doping with rGO (e.g., it decreases the resistivity by some 15%), but rather from the content of P_2_O_5_. A ratio of 10/90 of P_2_O_5_/ZnO decreases the resistivity from about 700 Ωm in sample ZnOrGO down to about 110 Ωm (e.g., almost seven-fold) in sample 90ZnO10 P_2_O_5_rGO. A further increase in the P_2_O_5_ content does not further decrease the resistivity.

## 4. Conclusions

Nanostructured films based on ZnO, P_2_O_5_, and rGO were successfully synthesized via the one-step sol–gel method. XRD patterns of analyzed samples showed that in films containing P_2_O_5_, a hexagonal phase of ZnO was identified, with the crystallite size decreasing with increasing P_2_O_5_/ZnO molar ratios from 10/90 (18 nm) to 20/80 (10 nm), respectively. The SEM investigations were in agreement: the distribution of crystallite diameters of 5–10 nm for samples ZnO, ZnOrGO and 80ZnO20P_2_O_5_rGO, of 20 nm for 86/14 (molar ratio) ZnO/P_2_O_5_ and of 26 nm for 90/10 (molar ratio) ZnO/P_2_O_5_. FTIR and Raman-collected spectra confirmed the presence of ZnO, P_2_O_5_ and rGO. The band gaps, as estimated from the UVVISNIR investigations, decreased as a result of the rGO and phosphorous oxide contents with a minimum of 3.77 eV for the 20-layered 90ZnO10P_2_O_5_rGO film. The optical band gap (Eg) was found to be dependent on the number of layers and P_2_O_5_ content. Our prepared 10 mol % of P_2_O_5_ and 1 wt.% rGO in the 90ZnOrGO10P_2_O_5_ sample tuned the fluorescence properties. The enhanced near-band-edge (NBE) intensity, together with a quenching of the visible emissions by passivating the surface defects with simultaneous enhancement of conductivity of ZnO, was achieved within one sol–gel stage of synthesis. A doping with 1 wt. % rGO decreased the film’s resistivity by about 15%, while a molar ratio of 10/90 of P_2_O_5_/ZnO decreased the resistivity almost seven-fold, as identified from the electrical measurements performed at room temperature.

## Figures and Tables

**Table 1:** Sample denomination and compositions in precursors’ solutions.

Sample Denomination	ZnO/P_2_O_5_ % Molar Ratio	rGO wt.% as Respect to ZnO in Precursor Solution	Number of Layers
ZnO	100/0		1, 10, 20
ZnOrGO	100/0	1	1, 10, 20
80ZnO20P_2_O_5_rGO	80/20	1	1, 10, 20
86ZnO14P_2_O_5_rGO	86/14	1	1, 10, 20
90ZnO10P_2_O_5_rGO	90/10	1	1, 10, 20

**Table 2:** FTIR absorption band assignments.

Band Assignments	Glass	ZnO	ZnOrGO	90ZnO10P_2_O_5_ rGO	86ZnO14P_2_O_5_ rGO	80ZnO20P_2_O_5_ rGO
	FTIR Peaks (cm^−1^)					
Zn–O stretching mode [26]	-	546	546	546	546	546
υs (Si–O–Si) [27]	766	755	754	752	752	752
υs (Si-O-) [27]	906	901 988	896	898	898	895
(980–1050 cm^−1^) υs Si-O-P [29,30] (1050 cm^−1^) C-O-C stretching (epoxy group) [28]	-	-	1010	1008	1009	1011

**Table 3:** Geometrical parameters and average resistance and resistivity values of the investigated films.

Sample	Average Resistance (GΩ)	*w* (mm)	Average *l* (mm)	Resistivity (Ωm)
ZnO	2.1 (1)	2.6 (1)	1.3 (1)	820 (10)
ZnOrGO	0.78 (5)	3.2 (1)	0.7 (1)	700 (10)
90ZnO10P_2_O_5_rGO	0.11 (2)	3.2 (1)	0.6 (1)	110 (10)
80ZnO20P_2_O_5_rGO	0.12 (2)	3.5 (1)	0.8 (1)	120 (10)

## Data Availability

Not applicable.
